# Molecular Characterization of the Dual Effect of the GPER Agonist G-1 in Glioblastoma

**DOI:** 10.3390/ijms232214309

**Published:** 2022-11-18

**Authors:** Alex Hirtz, Yann Bailly, Fabien Rech, Julien Pierson, Hélène Dumond, Hélène Dubois-Pot-Schneider

**Affiliations:** 1 Université de Lorraine, CNRS, CRAN, F-54000 Nancy, France; 2 Université de Lorraine, CHRU-Nancy, Service de Neurochirurgie, F-54000 Nancy, France

**Keywords:** glioma, GPER agonist G-1, transcriptomic analysis, lipid metabolism, tubulin inhibitor, xenograft

## Abstract

Glioblastoma (GBM) is the most common primary brain tumor in adults. Despite conventional treatment, consisting of a chirurgical resection followed by concomitant radio–chemotherapy, the 5-year survival rate is less than 5%. Few risk factors are clearly identified, but women are 1.4-fold less affected than men, suggesting that hormone and particularly estrogen signaling could have protective properties. Indeed, a high GPER1 (G-protein-coupled estrogen receptor) expression is associated with better survival, especially in women who produce a greater amount of estrogen. Therefore, we addressed the anti-tumor effect of the GPER agonist G-1 in vivo and characterized its molecular mechanism of action in vitro. First, the antiproliferative effect of G-1 was confirmed in a model of xenografted nude mice. A transcriptome analysis of GBM cells exposed to G-1 was performed, followed by functional analysis of the differentially expressed genes. Lipid and steroid synthesis pathways as well as cell division processes were both affected by G-1, depending on the dose and duration of the treatment. ANGPTL4, the first marker of G-1 exposure in GBM, was identified and validated in primary GBM cells and patient samples. These data strongly support the potential of G-1 as a promising chemotherapeutic compound for the treatment of GBM.

## 1. Introduction

Gliomas are a large family of primary brain tumors whose classification is regularly updated. Since 2021, they are divided into three subtypes, characterized by their cell of origin combined with their molecular profiling: IDH mutated/1p19q codeleted oligodendroglioma, IDH mutated astrocytoma, or IDH wild-type glioblastoma (GBM). Four grades were also described, according to the severity of the tumor and the prognosis of the patients (WHO 2021) [1]. With the worst prognosis and an incidence of 3.2/100,000, GBM is always classified as Grade 4 [2].

The standard treatment of GBM is a surgical resection as wide as possible, followed by concomitant temozolomide (TMZ)-based chemotherapy and radiotherapy. Despite this burdensome treatment, recurrences occur within months after surgery from the resection margins. Few risk factors are known but men are more affected than women, with a sex ratio of 1.4:1. A hypothesis is that the estrogens present in greater amount in women have neuroprotective effects against GBM. Recently, Hirtz et al. explored the impact of estrogen biosynthetic enzyme or receptor expressions on glioma patient survival [3,4]. They showed that a high expression of the membrane G-protein-coupled estrogen receptor (GPER) was associated with better survival in IDHmut Grades 3 and 4 astrocytoma and GBM.

In humans, GPER is expressed in many organs, including the brain [5,6,7,8,9,10]. In the embryo, the absence of GPER alters the brain morphology due to poor development of sensory and motor neurons [11]. In adults, GPER is expressed in many brain regions [9,10], especially in several hypothalamic nuclei, such as the paraventricular, supraoptic, arcuate, and suprachiasmatic nuclei [12,13]. At the cellular level, GPER has been detected in neurons, astrocytes, and oligodendrocytes of the rat hypothalamus [14]. GPER is associated with the occurrence of migraine; it is a regulator of cognitive function, particularly in post-menopausal women, and involved in the neuroprotective effects of 17β-estradiol (E2) [15]. Taken together, these data suggest that the expression and/or functionality of GPER is essential for brain physiology.

GPER is a member of the Class A or “rhodopsin-like” GPCRs, subtype Gα_s_. Although the validation of a direct interaction of E2 with GPER is still controversial [16], E2 is commonly accepted as the endogenous ligand of GPER [17,18]. Hirtz and colleagues also characterized the impact of GPER activation in GBM cells exposed to the GPER ligand G-1 (GPR30-specific compound 1), identified as the first specific agonist for GPER in 2006 [4,19]. In vitro, G-1 triggered cell cycle arrest in the G2/M phase and subsequent transient cell proliferation arrest in U251 and LN229 GBM cells exposed to 1 µM for 72 h, due to a disruption of the microtubule assembly. Pretreatment of cells with GPER antagonists G-15 or G-36 did not alleviate G-1 effects, suggesting that they were, at least in part, independent of GPER.

In the present study, we confirmed the antiproliferative effect of G-1 in a model of GBM xenografted nude mice and aimed to clarify the G-1 mechanisms of action in GBM cells. We performed a transcriptomic analysis of U251 cells exposed to 1 µM or the IC50 dose of G-1 for 24 h or 72 h. Functional analysis of the differentially expressed genes revealed dose- and time-dependent effects of G-1 treatment: the lipid synthesis-related pathway was mainly altered by the IC50 dose at the earliest time of treatment, whereas cell division was affected by the 1 µM G-1 treatment regardless of the duration of the treatment. Signatures of G-1 exposure were then validated in the primary GBM cells. Our analysis identified for the first time a marker of G-1 exposure, ANGPTL4, which was validated in the GBM patient samples. Taken together, these data strongly support the potential of G-1 as a promising chemotherapeutic compound for the treatment of GBM.

## 2. Results

### 2.1. G-1 Elicits an Anti-Tumor Effect in GBM Cell Xenografted Nude Mice

**Figure 1:**
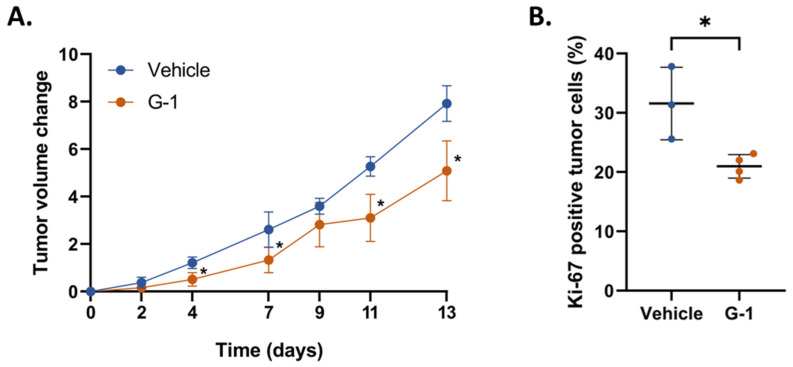
G-1 significantly decreases tumor growth in nude mice. (**A**) U87MG cells were subcutaneously injected into nude mice. Tumor volume change (TVC: for each mouse, the difference between tumor volume at each point of the experiment and tumor volume at the time of animal randomization) was measured during 2 weeks of treatment every 2 days. (**B**) Quantification of Ki-67-positive cells in tumors of the vehicle or G-1-treated mice. The data are presented as the mean ± SD. Significance level was determined using *t*-tests, with * indicating *p* < 0.05. Vehicle, *n* = 3; and G-1, *n* = 4.

Recently, Hirtz et al. demonstrated in vitro that G-1 triggered a cell proliferation arrest in U251 and LN229 GBM cells [4]. Therefore, we addressed the anti-tumoral effect of this GPER-agonist in a third GBM cell line, U87MG (Supplementary Figure S1), and in a corresponding model of female nude mice xenografted subcutaneously with U87MG GBM cells. When the tumor volume reached an average volume of 150 ± 40 mm^3^, mice were injected intra-peritoneally with G-1 or the corresponding vehicle (see Materials and Methods for details). Treatment was repeated 3 times a week until the heterotopic tumor volume reached the endpoint criterion. Tumor volume, but not animal weight, was significantly reduced in G-1 treated mice at Days 4, 7, 11, and 13, suggesting that G-1 might elicit anti-tumor processes in vivo (Figure 1A and Supplementary Figure S2). In line, we observed 34% less Ki-67 immunostaining in G-1-exposed tumors than in vehicle ones, whereas no difference in tumor histology was detected (Figure 1B and Supplementary Figure S3).

### 2.2. Transcriptomic Analysis of U251 GBM Cells Exposed to G-1

**Figure 2:**
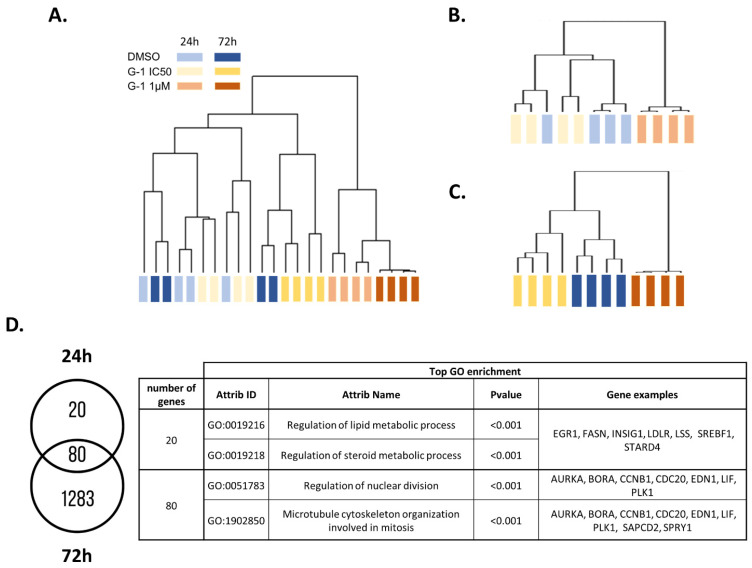
Transcriptomic profile of U251 cells exposed to G-1 in a dose- and time-dependent manner. (**A**) Hierarchical clustering of U251 GBM cells depending on the gene transcription between DMSO 0.01% (vehicle), G-1 IC50 (644.8 nM), and G-1 1 µM at 24 h and 72 h of treatment. (**B**) Hierarchical clustering at 24 h and (**C**) 72 h after DMSO 0.01%, G-1 IC50, and G-1 1 µM exposure. (**D**) Venn diagram of the corresponding differentially expressed genes after G-1 exposure at 24 h and 72 h. The corresponding top GO enrichments and genes associated are summarized in the table.

To evaluate the molecular mechanisms induced by G-1 treatment in GBM cells, U251 cells were exposed to 1 µM or the IC50 dose (644.8 nM) of G-1 for 24 h or 72 h (Supplementary Figure S4). Then, a comprehensive gene expression analysis was performed. By a one-way ANOVA, we identified 1653 deregulated genes (*p* < 0.05, Absolute Fold Change (|FC|) > 2), at least in one condition of treatment (Table S1). The hierarchical clustering of the differentially expressed genes (DEGs) revealed two main branches in the dendrogram, dividing the samples according to treatments: DMSO 0.01%/G-1 IC50 in one branch and G-1 1 µM in the other (Figure 2A). Cluster 1 was divided into two branches, driven mainly by G-1 IC50 72 h, whereas the G-1 IC50-treated cells during 24 h clustered with the DMSO controls. This subdivision probably indicated that the impact of the G-1 treatment at IC50 for 24 h was very weak. Thus, treatment with G-1 at 1 µM had the greatest effect, regardless of the duration of treatment.

To better evaluate the impact of treatment duration, we analyzed the data obtained after either 24 h or 72 h G-1 exposure. By a one-way ANOVA, we identified 100 and 1363 DEGs (*p* < 0.05, |FC| > 2; Table S2 and Table S3 respectively) in at least one condition after 24 h or 72 h of treatment, respectively. In both cases, the hierarchical clustering of the DEGs highlighted two main branches of the dendrogram, dividing the samples according to treatments: DMSO 0.01%/G-1 IC50 in one branch and G-1 1 µM in the other (Figure 2B,C). Interestingly, 80% of the DEGs identified at 24 h were also differentially expressed at 72 h and the top enriched GO terms were mainly associated with cell division. At 24 h, the 20 specific genes were linked to sterol and lipid metabolism, as suggested by upregulation of *SREBF1*, *LDLR*, *INSIG1*, *STARD4*, *LSS*, *EGR1*, and *FASN* (Figure 2D).

### 2.3. Identification and Validation of ANGPTL4 Expression as a Marker of G-1 Exposure

**Figure 3:**
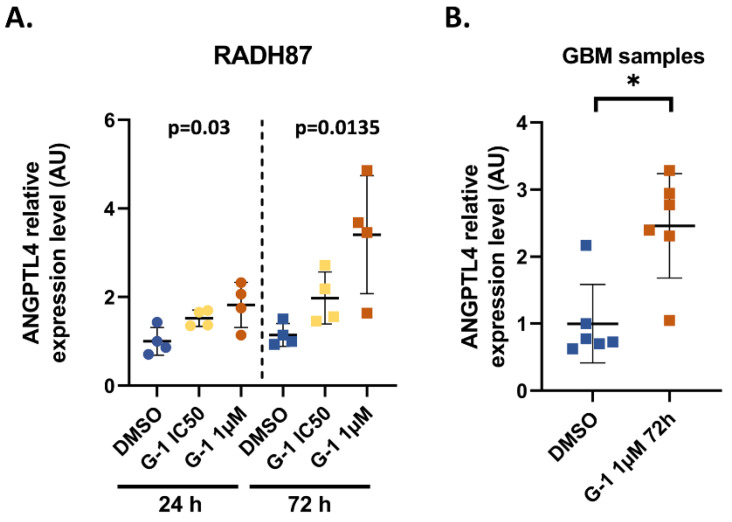
ANGPTL4 is a marker of G-1 exposure. (**A**) Expression level of *ANGPTL4* determined by RT-qPCR in RADH87 primary GBM cells after exposure to DMSO 0.01% (vehicle) or G-1 IC50 (562.5 nM)/1 µM for 24 h and 72 h; *n* = 4. (**B**) Expression level of *ANGPTL4* in 6 GBM patient samples after exposure to vehicle or G-1 1 µM after 72 h. Data are presented as the mean ± SD. Significance level was determined using ANOVA for RADH87 and by Wilcoxon tests in patient samples, where * indicates *p* < 0.05.

Transcriptome analysis revealed that *ANGPTL4* was the most deregulated gene after G-1 treatment for 24 h and 72 h, by 38-fold and 231-fold, respectively. This result obtained from U251 cells was verified in RADH87 primary GBM cells exposed to G-1 for 24 h or 72 h. RT-qPCR data confirmed that *ANGPTL4* was also significantly upregulated in RADH87 primary GBM cells (by 1.8-fold and 3-fold at 24 h and 72 h, respectively) as well as in patient tumors (by 2.5-fold) treated by 1 µM G-1 for 72 h (Figure 3A,B). Therefore, *ANGPTL4* might be considered as the first marker of G-1 exposure in GBM.

### 2.4. G-1 Impairs Lipid Metabolism and Nuclear Division Pathways

**Figure 4:**
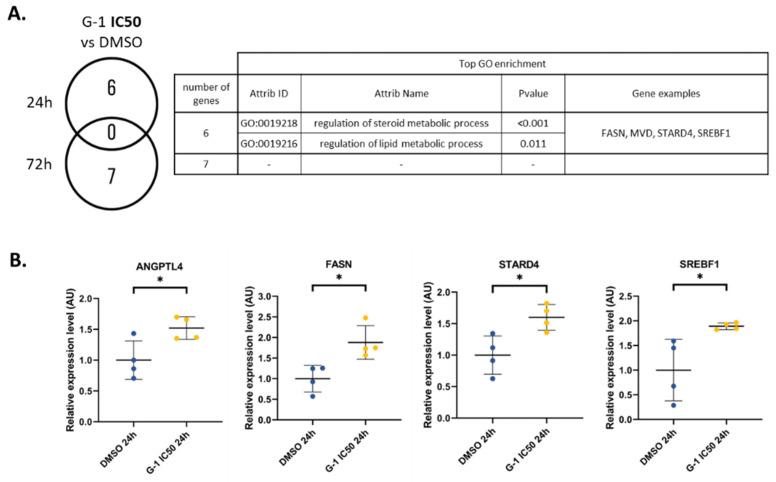
Dual effect of G-1 altering lipid metabolism and nuclear division pathways. (**A**) Number of vehicle versus G-1 IC50 (644.8 nM) DEGs and table of associated functions at 24 h and 72 h. (**B**) RT-qPCR analysis of the *FASN*, *STARD4*, *ANGPTL4*, and *SREBF1* expressions in the RADH87 primary GBM cell line after 24 h of G-1 IC50 (562.5 nM) exposure. Data are presented as the mean ± SD. Significance level was determined using *t*-tests, where * indicates *p* < 0.05; *n* = 4.

To decipher the real impact of the G-1 dose, we then performed two-by-two comparisons (unpaired *t*-test *p* < 0.05, |FC| > 2). At a dose of G-1 IC50, only 6 and 7 genes were significantly deregulated after 24 h and 72 h, respectively (Tables S4 and S5). Associated functions were related to lipid/steroid metabolic processes, as suggested by the upregulation of *FASN*, *MVD*, *STARD4*, and *SREBF1* at 24 h. At 72 h, even if no function was significantly enriched, among the seven DEGs, three were related to lipid metabolism, such as *ACSBG1*, *MGLL*, and *ANGPTL4* (Figure 4A). The significant upregulation of *FASN*, *ANGPTL4*, *STARD4*, and *SREBF1* was confirmed in RADH87 primary GBM cells, by 1.9-fold, 1.5-fold, 1.6-fold, and 1.9-fold, respectively (Figure 4B).

**Figure 5:**
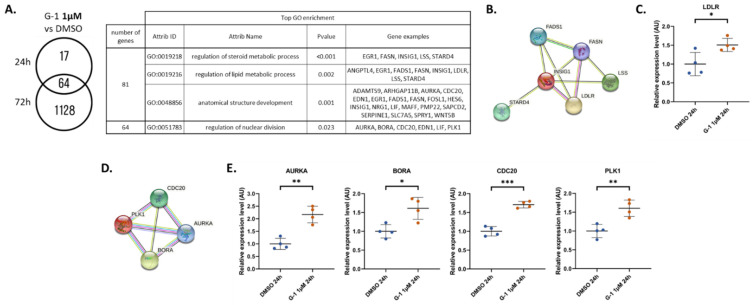
(**A**) Number of vehicle versus G-1 1 µM DEGs and table of associated functions at 24 h and 72 h. (**B**) Protein–protein interactions from the STRING interaction database. Among the 17 DEGs selected after 24 h exposure to G-1 1 µM, the cluster FADS1, FASN, LSS, LDLR, INSIG1, and STARD4 was identified. (**C**) RT-qPCR analysis of *LDLR* expression in the RADH87 primary GBM cell line after 24 h of G-1 1 µM exposure (**D**) Protein–protein interactions from the STRING interaction database. Among the 64 common DEGs selected after 24 h and 72 h exposure to G-1 1 µM, the cluster CDC20, PLK1, AURKA, and BORA was identified. (**E**) RT-qPCR analysis of *PLK1*, *BORA*, *AURKA*, and *CDC20* expressions in the RADH87 primary GBM cell line after 24 h of G-1 1 µM exposure. Data are presented as the mean ± SD. Significance level was determined using *t*-tests, where *** indicates *p* < 0.001, ** indicates *p* < 0.01, and * indicates *p* < 0.05; *n* = 4.

For the 1 µM G-1 treatment, we highlighted a higher number of deregulated genes (Figure 5A). After 24 h of G-1 treatment, 81 genes were differently expressed (Table S6). Among them, no specific enrichment was found for the 37 downregulated genes. In contrast, the 44 upregulated genes were associated with regulation of steroid and lipid metabolic processes or anatomical structure development. All these functions were driven by *INSIG1*, *FASN*, *FADS1*, *LSS*, and *LDLR* (Figure 5B). Interestingly, these five genes were specifically upregulated at 24 h but not at 72 h. Upregulation of LDLR by G-1 1 µM was confirmed in RADH87 cells (Figure 5C). Sixty-four genes were deregulated by G-1 for the two treatment durations. These genes were linked to the GO term “regulation of nuclear division”, as suggested by the upregulation of genes encoding the key interacting proteins AURKA, BORA, CDC20, and PLK1 (Figure 5D). AURKA and PLK1 are two serine-threonine protein kinases involved in regulation of cell division and microtubule protein phosphorylation. BORA is the coactivator of AURKA. The differential expression of this functional gene cluster was fully confirmed in RADH87 cells (Figure 5E).

**Figure 6:**
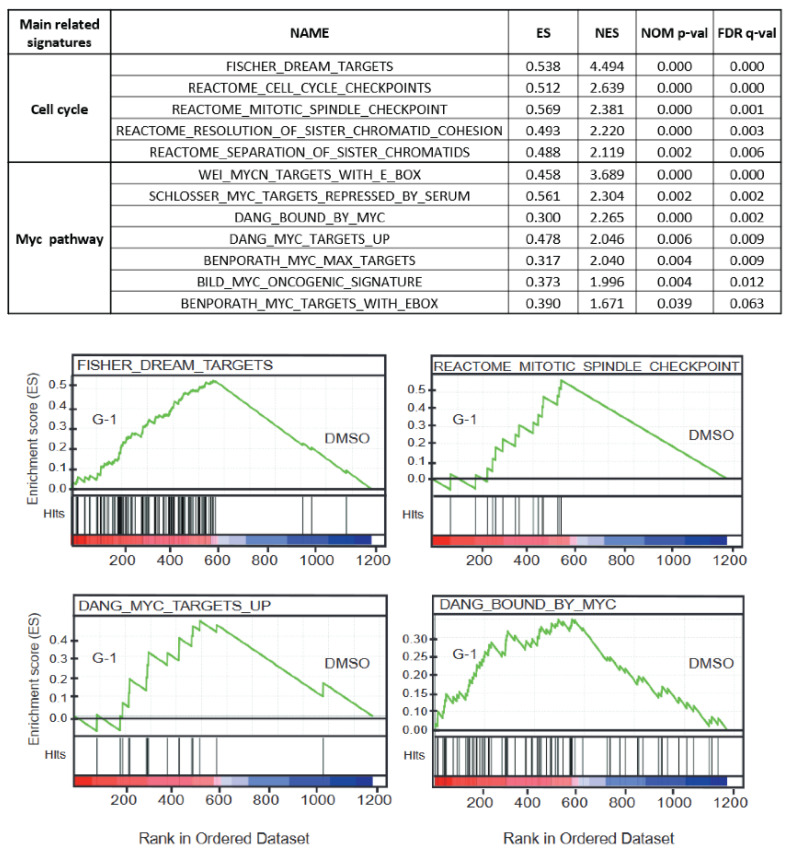
GSEA signature enrichment of G-1 is mainly related to the cell cycle and Myc pathways. GSEA analysis using the gene expression profile of U251 cells exposed to 1 µM of G-1 for 72 h showed significant positive enrichment for gene sets that are linked to mitotic spindle and chromatid separation and gene sets related to Myc pathway signatures.

In total, 1192 DEGs were identified after 72 h of 1 µM G-1 treatment (Table S7). No biological function was significantly enriched in this dataset when separating the 585 up- and 607 downregulated genes. As this total number of DEGs is not compatible with a pathway analysis, we performed an unsupervised GSEA signature enrichment (Figure 6). We identified a significant increase in several signatures, mainly related to cell cycle regulation. Among them are the reactomes “MITOTIC_SPINDLE_CHECKPOINT” or “SEPARATION_OF_SISTER_CHROMATIDS”. Several Myc signatures were also enriched as “DANG_MYC_TARGETS_UP” or “DANG_BOUND_BY_MYC.”

### 2.5. The Molecular G-1 Signature Is Similar to Microtubule Targeting Agent One

To further characterize the role of G-1 treatment (1 µM, 72 h), we used a connectivity map (CMap) approach. Only compounds with a connectivity score of 90 or higher were selected. Interestingly, among the 17 ranked molecules, we identified 9 tubulin inhibitors, supporting the role of G-1 as a molecule acting on the cell cycle and particularly on tubulin polymerization (Table 1). We also highlighted two PKC (protein kinase C) activators affecting cell cycle progression and a PLK (polo-like kinase) inhibitor known to cause mitotic blockage and apoptotic cell death.

## 3. Discussion

In a previous study performed in vitro on U251 and LN229 GBM cell lines, Hirtz et al. showed that a 1 µM G-1 exposure caused a decrease in proliferation characterized by altered tubulin polymerization and cell cycle arrest in the G2/M phase [4]. These results were confirmed in U87MG and consistent with those found in other cell models [20,21]. In the present in vivo study, U87MG GBM cells were grafted heterotopically into nude mice. As indicated by Sharma et al., G-1 administration did not impair weight gain in intact female mice [22]. We observed an antitumor effect of G-1 in vivo, with a significant decrease in tumor size and Ki-67 immunostaining. This suggested once again that the cellular response initiated by G-1 might elicit a beneficial effect against tumor growth. Nevertheless, the proof of concept that G-1 may be used as a chemotherapeutic compound against GBM has to be confirmed in orthotopic xenograft models to ensure tumor cells to interact with the cerebral microenvironment and address G-1 brain–blood-barrier crossing. Camphausen et al. showed that while the U87MG and U251 gene expression profiles appear quite disparate in a monolayer culture in vitro, these differences are attenuated in sub-cutaneous xenografts and almost disappear in intra-cranial xenografts [23]. Therefore, such models also could be relevant to validate the G-1-dependent signaling processes and downstream target genes identified below.

As a follow-up to previous work from Hirtz et al., we went back in vitro to characterize in detail the mechanisms of action of G-1 by treating U251 GBM cells at two doses (IC50, 1 µM) and at two different times (24 h and 72 h) [4]. This refinement of the experimental conditions allowed us to detect two distinct antiproliferative processes triggered by G-1.

At IC50, G-1 disrupted lipid metabolism. It increased the expression of a set of genes, including *ACSBG1*, *ANGPTL4*, *FASN*, *MVD*, *MGLL*, *SREBF1*, and *STARD4*, that are involved in this process. Among them, ACSBG1, FASN, and MGLL are critical enzymes related to fatty acid (FA) metabolism. The role of G-1 treatment on lipid metabolism, especially FA metabolism, has recently been demonstrated in vivo [22]. Authors show that G-1 enhances FA oxidation by upregulating FA oxidation enzymes. In U251 cells, G-1 treatment leads to an increase in Acyl-CoA Synthetase Bubblegum Family Member 1 (ACSBG1), an enzyme that activates FA for both degradation via β-oxidation or synthesis of cellular lipids [24]. Similarly, we showed an upregulation of both MGLL, the monoacylglycerol lipase that coverts triglycerides into free FA, and FASN, the main enzyme of the palmitic acid (PA) synthesis. PA is the precursor for the synthesis of phospholipids, triglycerides (TG), cholesterol esters, and protein acetylation. An increase in FASN expression has been reported as a poor prognosis, since its expression correlates with the grade of glioma [25,26]. Similarly, stimulation of lipid metabolism fuels energetic metabolism in cancer cells and contributes to their proliferation [27]. Conversely, we found here that stimulation of this signaling pathway by G-1 is concomitant with decreased proliferation of GBM cells. One hypothesis is that increased FASN expression promotes free PA production into the cells, which has been described to decrease cell viability [28]. In line, excess PA has been shown to be cytotoxic to neural stem cells [29]. The effects of PA depend on the balance between oleic acid (OA) and PA production. For example, in the breast cancer cell line MDA-MB-231, exposure to OA increases, whereas PA decreases their proliferation and induces apoptosis by inhibiting PI3K [30,31]. In GBM cells treated with G-1, the expression of FASN is increased, but not that of steryl CoA desaturase 1, which produces OA. It is therefore likely that PA production is higher than OA production, which might contribute to prevent cell proliferation.

In parallel, *ANGPTL4* expression is significantly induced regardless of the dose and duration of G-1 treatment of U251 cells. Even to a lesser extent, this induction was confirmed in RADH87 primary GBM cells, as well as in patient samples exposed to G-1 1 µM for 72 h in ex vivo culture. ANGPTL4 is an adipokine, a member of the angiopoietin-like protein family involved in angiogenesis regulation, lipid metabolism, cancer progression, and metastasis. Tsai et al. showed in U87MG cells that overexpression of ANGPTL4 stimulated TMZ resistance and cancer stemness via a positive feedback loop involving the EGFR/PI3K/Akt/4E-BP1 signaling pathway [32]. In U251 cells, G-1 treatment activated ANGPTL4 expression but acted synergistically with TMZ to prevent proliferation [4]. G-1 can activate EGFR indirectly via Src phosphorylation and HB-EGF release [33]. In the presence of G-1, competition between EGFR activation by HB-EGF and ANGPTL4 may alleviate the development of TMZ resistance and even decrease cell proliferation. Indeed, ANGPTL4 expression correlated with a decrease in cell proliferation in a model of osteosarcoma by limiting aberrant branched-chain amino acid metabolism, which is often linked to cancer progression and invasion [34]. Previous data also suggested that ANGPTL4 limited tumor aggressiveness and invasiveness by preventing angiogenesis and vascular permeability [35,36]. While its precise role in GBM remains to be established, in relation to G-1-initiated signaling, increased ANGPTL4 expression appears to be a robust marker of exposure to this GPER agonist, especially in U251 cells. Since ANGPTL4 is regulated by several transcription factors at the crossroad of metabolism and tumor development, the signaling pathway leading to its upregulation after G-1 exposure remains to be determined [37].

Using our transcriptomic approach, we also demonstrated that treatment with 1 µM G-1 for 72 h increased the expression of several actors of centrosome separation that participate in the cell cycle transition to the G2/M phase. These data were confirmed in RADH87 primary GBM cells. The key regulator of this signaling pathway is PLK1. In GBM cells, a reduction in PLK1 levels by siRNA resulted in a decrease in cell proliferation [38]. However, overexpression of PLK1 can also lead to defects in chromosome segregation and abnormal cytokinesis, generating plurinucleated cells with reduced proliferative potential [39]. In agreement, several cell cycle checkpoints and Myc signatures were enriched by G-1 treatment for 72 h. Myc is a transcription factor that regulates the transcription of multiple mitotic spindle genes, leading to impaired mitotic spindle formation [40].

At the cellular level, Hirtz et al. confirmed that G-1 disrupted the cell division, in particular tubulin polymerization, and observed plurinucleate cells [4]. In accordance, the CMap approach confirmed that G-1 might act like tubulin-destabilizing compounds. Most of them are tubulin depolymerization agents, such as vinca-alkaloids, including vinorelbine, vincristine, vinblastine, and vindesine, or NPI-2358 [41,42]. Vinca-alkaloids were previously shown to improve the treatment of some GBM patients [43]. Furthermore, the molecular signature of the G-1 treatment (1 µM, 72 h) is very similar to that of nocodazole, which disrupts microtubules. Lv et al. have already observed a similar impact of G-1 and nocodazole in two breast cancer cell lines [44]. We also identified ABT-751, flubendazole, and mebendazole, which target the colchicine-binding site on the tubulin [45]. Mebendazole disrupts microtubule formation in GBM cells, and its activity correlates with reduced tubulin polymerization in vitro. Moreover, mebendazole increases the TMZ effect in orthotopic xenografted mouse models of glioma [46]. Altogether, these data confirmed that G-1 could be a promising microtubule-targeting agent. Interestingly, we also highlighted two PKC activators, prostratin and ingenol, that displayed a similar signature to G-1. In contrast to other tumors, it has been shown that the use of PKC activators, targeting specific PKC isoenzymes, could be promising for GBM treatment [47].

Taken together, these data make G-1 an effective anti-proliferative molecule, which might be used for anti-tumor therapy in GBM. However, we were unable to confirm that the G-1 effects are dependent on the presence of GPER, since no signature associated with GPCRs were retrieved.

One of the major advantages of using G-1 in the context of GBM treatment might be the absence of side effects observed in animals [22]. A phase I/IIA clinical trial (accession number: NCT04130516) initiated for the treatment of advanced melanoma will also provide information on potential toxicity and patient metabolic response to orally administered G-1. In the present study, G-1 elicited two distinct antiproliferative effects that might synergize with TMZ: by disrupting lipid metabolism and by targeting microtubules. Once the dosage, distribution, and pharmacokinetics of G-1 in GBM and the surrounding brain tissue will be elucidated, G-1 should be considered, alone or in combination, for the management of GBM patients.

## 4. Materials and Methods

### 4.1. Cell Culture and Treatment

The U87MG GBM cell line was purchased from American Type Culture Collection (Manassas, VA, USA) and the U251 GBM cells were obtained from Sigma-Aldrich (Saint-Quentin-Fallavier, France). The RADH87 primary GBM cell line was kindly provided by Dr. Tony Avril (Inserm U1242, University of Rennes, Centre E. Marquis, Rennes, France). All cell lines were routinely cultured in DMEM (Dulbecco’s modified eagle medium), phenol red-free (Gibco) supplemented with 10% decomplemented FBS, 1% essential amino acids (Sigma-Aldrich), 0.5% non-essential amino acids (Sigma-Aldrich), 0.4% vitamins (Sigma-Aldrich), 1.25% sodium pyruvate (Sigma-Aldrich), and 1% streptomycin/penicillin (Sigma-Aldrich) at 37 °C in a humidified 5% CO_2_ atmosphere. For each experiment, the cells were trypsinized, counted with a Thoma cell-counting chamber, seeded, and grown for 24 h. Then, the cells were deprived from steroid hormones for 24 h in 10% charcoal-stripped FBS medium. Thereafter, cells were exposed for 24 h or 72 h to 1 µM G-1 or the G-1 corresponding to the IC50 of each cell line (Figure S1). G-1 was purchased from Tocris-BioTechne (Noyal Chatillon sur Seiche, France).

### 4.2. Transcriptomic Analysis

mRNAs from 4 independent biological replicates of 3 conditions DMSO 0.01%, G-1 IC50 and G-1 1 µM and 2 durations of treatment (24 h and 72 h) were used to perform the microarray experiment. Genome-wide expression profiling was performed using the low-input QuickAmp labeling kit and human SurePrint G3 8 × 60 K pangenomic microarrays (Agilent Technologies, Santa Clara, CA, USA). Gene expression data were processed using Feature Extraction (Agilent Technologies). The data discussed in this publication have been deposited in NCBI’s Gene Expression Omnibus (GEO) and are accessible on 17 november 2022 through GEO Series accession number GSE214533 (https://www.ncbi.nlm.nih.gov/geo/query/acc.cgi?acc=GSE214533 20 October 2022). Statistical analyses were performed with GeneSpring GX software v11.5 (Agilent Technology, Santa Clara, CA, USA). Differentially expressed genes were identified by a one-way ANOVA test followed by a Tukey multiple-comparison (with a Benjamini FWER correction) test, with a *p*-value < 0.05 and an absolute fold change (|FC|) > 2. For 2-by-2 comparisons, a *t*-test with a *p*-value < 0.05 and an absolute fold change (|FC|) > 2 was done. Clustering analysis was performed using Cluster 3.0 and TreeView 1.6 (Java Treeview) using uncentered correlation and complete linkage.

### 4.3. Bioinformatic Analysis

Gene annotation was based on gene ontology and enrichment for specific biological functions was determined using the FuncAssociate 2.0 program (FuncAssociate: The Gene Set Functionator [48]). For interpretation of transcriptomic data, we performed a STRING protein–protein interaction (string-db.org). Gene set enrichment analysis (GSEA, Gene Set Enrichment Analysis) was used to check whether an a priori defined set of genes shows statistically significant, concordant differences between two biological states. GSEA was performed by using the Java tool developed at the Broad Institute (Cambridge, MA, USA). Unsupervised GSEA was performed with the whole C2 collection of curated gene sets from the molecular signatures database (MSigDB). The enrichment score (ES) was determined after 1000 permutations, as previously described [49,50].

### 4.4. GBM Tumor from Patients

Human GBM samples were collected from the neurosurgery department of the CHRU in Nancy. All patients gave informed consent (Collection reference n°DC-2019-3739). Patients’ characteristics are listed in Table S8. Six GBM patient samples were fragmented and treated for 72 h by DMSO 0.01% as vehicle or 1 µM G-1. After G-1 exposure, RNA extraction and RT-qPCR analyses were performed on each fragment from each patient tumor.

### 4.5. RT-qPCR

Total RNA was extracted from the cell and human tumor samples using the E.Z.N.A HP total RNA kit (Omega Biotek, Norcross, GA, USA), following the manufacturer’s instructions. Reverse transcription of 500 ng total RNA was performed according to the iScript Reverse Transcription Supermix for RT-qPCR kit (BioRad, Hercules, CA, USA). Real-time PCR analyses were performed using iTaq Universal SYBR Green Supermix (BioRad, Hercules, CA, USA) with CFX96 Real Time PCR Detection System and software (Biorad, Hercules, CA, USA). The primers used are listed in Table S9. Assays were performed at least in triplicate, and the mean values were used to calculate the expression levels, using the Starting Quantity (SQ) method, referring to the U6 housekeeping gene expression. When treatments were performed, the variation in expression was reported as treated/DMSO 0.01%-treated cells (vehicle).

### 4.6. Animal Xenograft Model and Treatments

Eight immunodeficient female mice (NMRI-nu, Janvier Labs, Le Genest-Saint-Isle, France) xenografted heterotopically with U87MG cells were used within the agreement of the French Minister of Research (agreement n°APAFiS #35594). U87MG cells were re-suspended in Hanks’ Balanced Salt Solution (Sigma Aldrich Life science ref H8264). The cells (2 × 10^6^ cells/100 μL) were injected subcutaneously into the right flank of 6-week-old nude mice. For tumor induction, mice were anesthetized by intraperitoneal injection (4 μL/g of weight) of a solution mixture of xylazine/ketamine (90 and 8 mg/kg, respectively). Tumor size was monitored every 2 days by measuring two dimensions (length and width) with a caliper and the volume was estimated by calculating the length × width^2^. Tumor growth was normalized by taking tumor volume at the start of treatment as a reference. When tumors reached an average volume of 150 ± 40 mm^3^, animals were randomly divided according to their tumoral volume into 2 groups. As the tumor volume of one mouse never reached this threshold, only 7 mice were treated. Two treatment groups were assigned: animals received G-1′s vehicle (*n* = 3) or G-1 molecule (*n* = 4) by intraperitoneal injection. For this, G-1 was diluted in 10% ethanol to a final concentration of 0.5 mg/mL. The treatment was performed every 2 days during 2 weeks of treatment (with a wash-out of 2 days during the weekend). Treatments were injected with a fixed volume of 300 µL. When the heterotopic tumor volume reached 1500 mm^3^ (endpoint criterion), the mice were sacrificed, and the tumors were harvested for further analyses.

### 4.7. Immunohistochemistry and Hematoxylin and Eosin (H&E) Staining

Formalin-fixed tumor tissues were embedded in paraffin and cut into 5 µM sections. The tissue sections were stained with H&E using standard procedures. For immunohistochemical analysis, tissue sections were deparaffinized in toluene and rehydrated through graded alcohols. Antigen retrieval was performed by heating the slides in sodium citrate buffer (pH 6.0). Ki-67 staining was performed using rabbit anti-Ki-67 antibody (Ab16667, Abcam, Cambridge, UK) with 1:1500 dilution at 4 °C overnight. Slides were then treated with 6% H_2_O_2_ for 20 min to quench endogenous peroxidase activity. Sections were then washed with PBST and incubated with simple stain mouse max PO ^®^ anti-rabbit (414341F, Nichirei, Tokyo, Japan) antibodies at 37 °C for 20 min. The number of Ki-67-positive cells was quantified automatically using the QuPath (v.02.3) software.

### 4.8. Connectivity Map

The clue.io platform (https://clue.io/query 20 October 2022) was used to compare the G-1 (1 µM, 72 h) signature to the collection of hundreds-of-thousands of L1000 gene-expression profiles from cells exposed to reference perturbagens, available in the public LINCS Connectivity Map (CMap) resource [51]. Perturbagens with the highest similarity with G-1 signature were identified based on the CMap connectivity score (tau). Only perturbagens with Tau of +90 or higher were selected in our study.

### 4.9. Statistical Analysis

Results are expressed as the means ± SD (standard deviation). Normal distribution of the data were evaluated by a Shapiro–Wilk test. Statistical significance was evaluated by Students’s *t*-test for two-by-two comparison for unpaired data and by a Wilcoxon test for paired data. Multiple comparisons were done using one-way analysis of variance (ANOVA). Standard deviations (SD) were indicated on the figures, as advocated by Altman and Bland [52]. The number of independent experiments performed was indicated as n in each figure legend.

## Figures and Tables

**Table 1:** The molecular signature of G-1 resembles to that of microtubule-targeting agents. The Cmap approach was used, allowing identification of the top molecules (tau > 90) whose molecular signatures are close to those of G-1 after 72 h of treatment with 1 µM of the U251 cell line.

Id	median_tau_score	Name	Belongs to
BRD-K91145395	98.86	prostratin	PKC activator
BRD-K12539581	98.48	nocodazole	tubulin inhibitor
BRD-K86003836	98.31	flubendazole	tubulin inhibitor
BRD-K10916986	98.31	vinorelbine	tubulin inhibitor
BRD-A76528577	98.10	vincristine	tubulin inhibitor
BRD-K77987382	97.63	mebendazole	tubulin inhibitor
BRD-K37456065	97.37	VU-0365114-2	M5 modulator
BRD-K91623615	97.33	ABT-751	tubulin inhibitor
BRD-K94325918	96.28	kinetin-riboside	antiproliferative and apoptosis inhibitor
BRD-A54927599	96.17	KF-38789	P-selectin-mediated cell adhesion inhibitor
BRD-K35687265	95.98	ON-01910	PLK inhibitor
BRD-A55594068	95.91	vinblastine	tubulin inhibitor
BRD-K59753975	95.71	vindesine	tubulin inhibitor
BRD-K36055864	94.89	cycloheximide	protein synthesis inhibitor
BRD-K01976263	94.67	emetine	Wnt/β-catenin inhibitor
BRD-A52650764	94.47	ingenol	PKC activator
BRD-K99498722	91.02	NPI-2358	tubulin inhibitor

## Data Availability

The data discussed in this publication have been deposited in NCBI’s Gene Expression Omnibus (GEO) and are accessible on 17 november 2022 through GEO Series accession number GSE214533 (https://www.ncbi.nlm.nih.gov/geo/query/acc.cgi?acc=GSE214533 20 October 2022).

## Supplementary Materials

The following supporting information can be downloaded at: https://www.mdpi.com/article/10.3390/ijms232214309/s1.
