# Supplementary material for: Molecular Characterization of the Dual Effect of the GPER Agonist G-1 in Glioblastoma
Source: Int J Mol Sci. 2022 Nov 18;23(22):14309. doi: 10.3390/ijms232214309 (PMC9695951; doi:10.3390/ijms232214309)
Supplement: Supplementary file 1 [file ijms-23-14309-s001.zip › ijms-2014588-supplementary.pdf]

Supplementary Materials: The following supporting information can be downloaded at: www.mdpi.com/xxx/s1, Figure S1: G-1 impact on U87MG cells in vitro. Figure S2: Animal weight follow-up during G-1 treatment;. Figure S3: Representatives Ki-67 and H&E staining of tumors of vehicle or G-1 treated mice; Figure S4: Half-maximal inhibitory concentration (IC50) determination of G-1 in U251 and RADH87 GBM cells.; Table S1: List of significant DEGs by G-1 treatment ($p < 0.05$, $|FC| > 2$) in at least one condition (G-1 IC50 24h, G-1 1µM 24h, G-1 IC50 72h, G-1 1µM 72h).; Table S2: List of significant DEGs by G-1 treatment ($p < 0.05$, $|FC| > 2$) in at least one condition (G-1 IC50 or G-1 1µM) at 24h.; Table S3: List of significant DEGs by G-1 treatment ($p < 0.05$, $|FC| > 2$) in at least one condition (G-1 IC50 or G-1 1µM) at 72h.; Table S4: List of significant DEGs by G-1 IC50 treatment ($p < 0.05$, $|FC| > 2$) at 24h.; Table S5: List of significant DEGs by G-1 IC50 treatment ($p < 0.05$, $|FC| > 2$) at 72h.; Table S6: List of significant DEGs by G-1 1µM treatment ($p < 0.05$, $|FC| > 2$) at 24h.; Table S7: List of significant DEGs by G-1 1µM treatment ($p < 0.05$, $|FC| > 2$) at 72h.; Table S8: Patients ‘characteristics; Table S9: Primer sequences used for RT-qPCR
