# Supplementary material for: Molecular Characterization of the Dual Effect of the GPER Agonist G-1 in Glioblastoma
Source: Int J Mol Sci. 2022 Nov 18;23(22):14309. doi: 10.3390/ijms232214309 (PMC9695951; doi:10.3390/ijms232214309)
Supplement: Supplementary file 1 [file ijms-23-14309-s001.zip › Supplementary M&M.pdf]

Supplementary Materials:

To assess the IC50 of G-1, GBM cells were seeded in 96-well plates in complete medium for 24h and then deprived from steroid hormones for another 24h in 10% charcoal-stripped FBS medium. Thereafter, cells were exposed for 72h to a range of 1nM to 10μM G-1 and the number of metabolically active cells was assessed by MTT assay. IC50 was calculated using GraphPad Prism 8.
