# Supplementary figures and images for: Molecular Characterization of the Dual Effect of the GPER Agonist G-1 in Glioblastoma
Source: Int J Mol Sci. 2022 Nov 18;23(22):14309. doi: 10.3390/ijms232214309 (PMC9695951; doi:10.3390/ijms232214309)

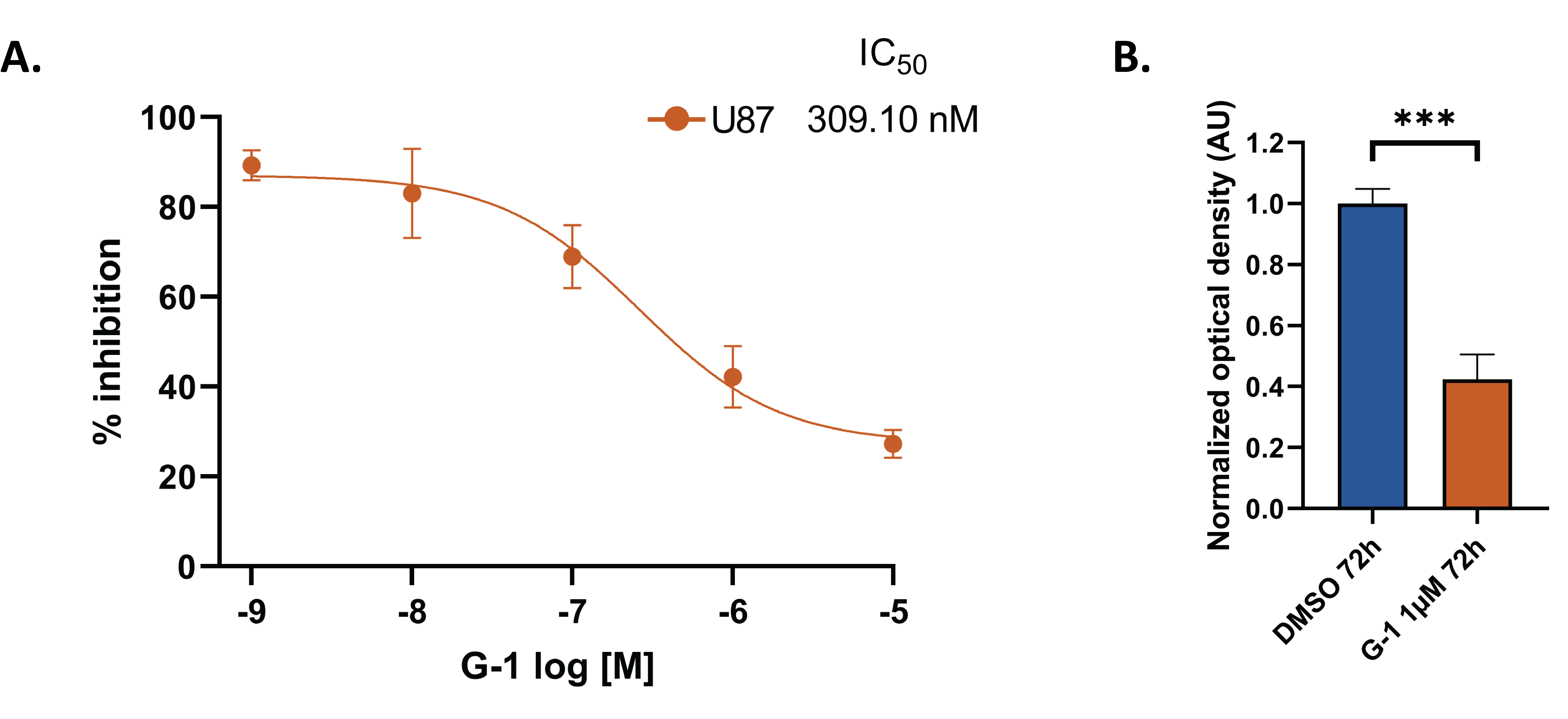

Supplement: Supplementary file 1 [file ijms-23-14309-s001.zip › Figure S1.png]

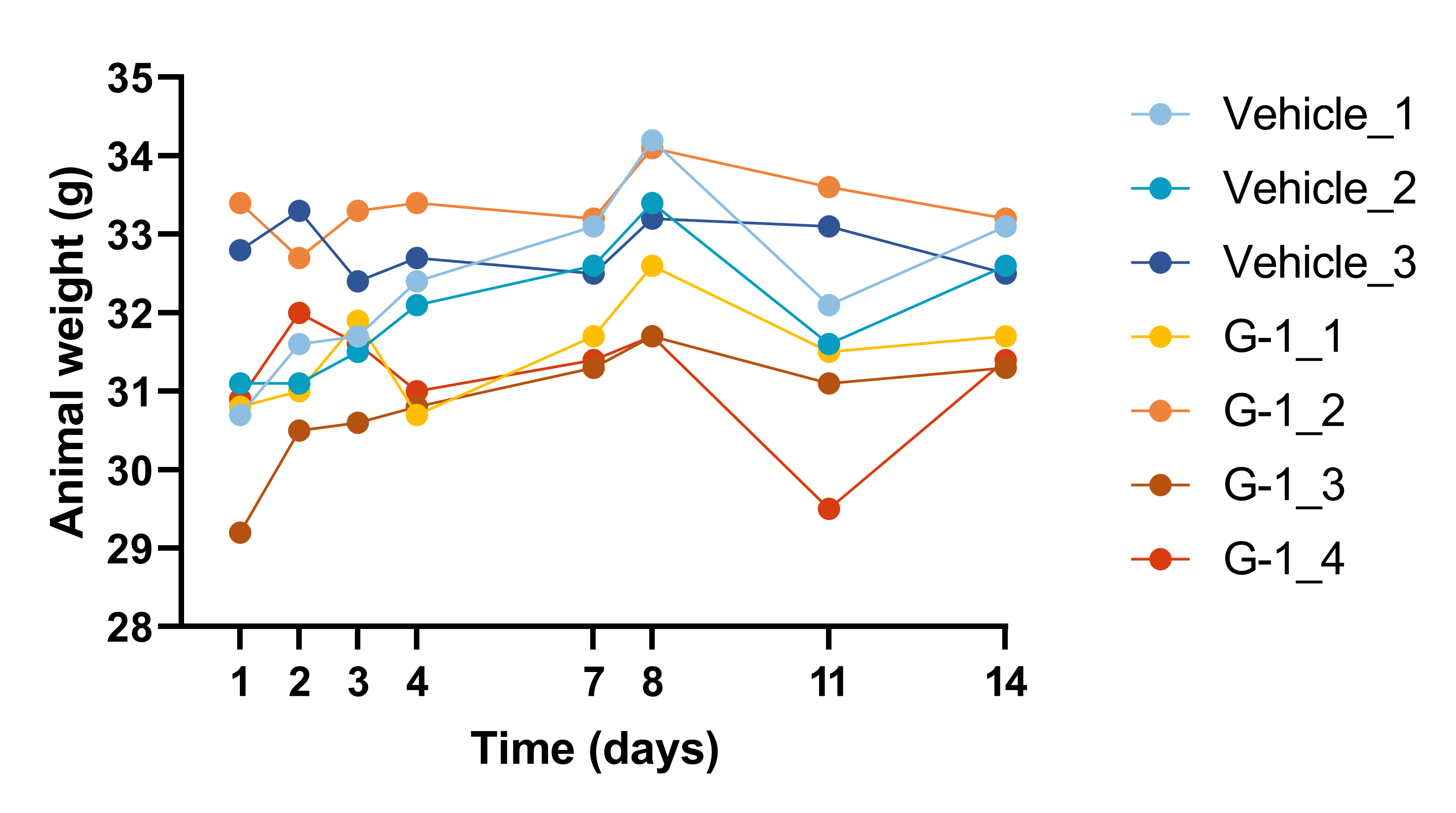

Supplement: Supplementary file 1 [file ijms-23-14309-s001.zip › Figure S2.png]

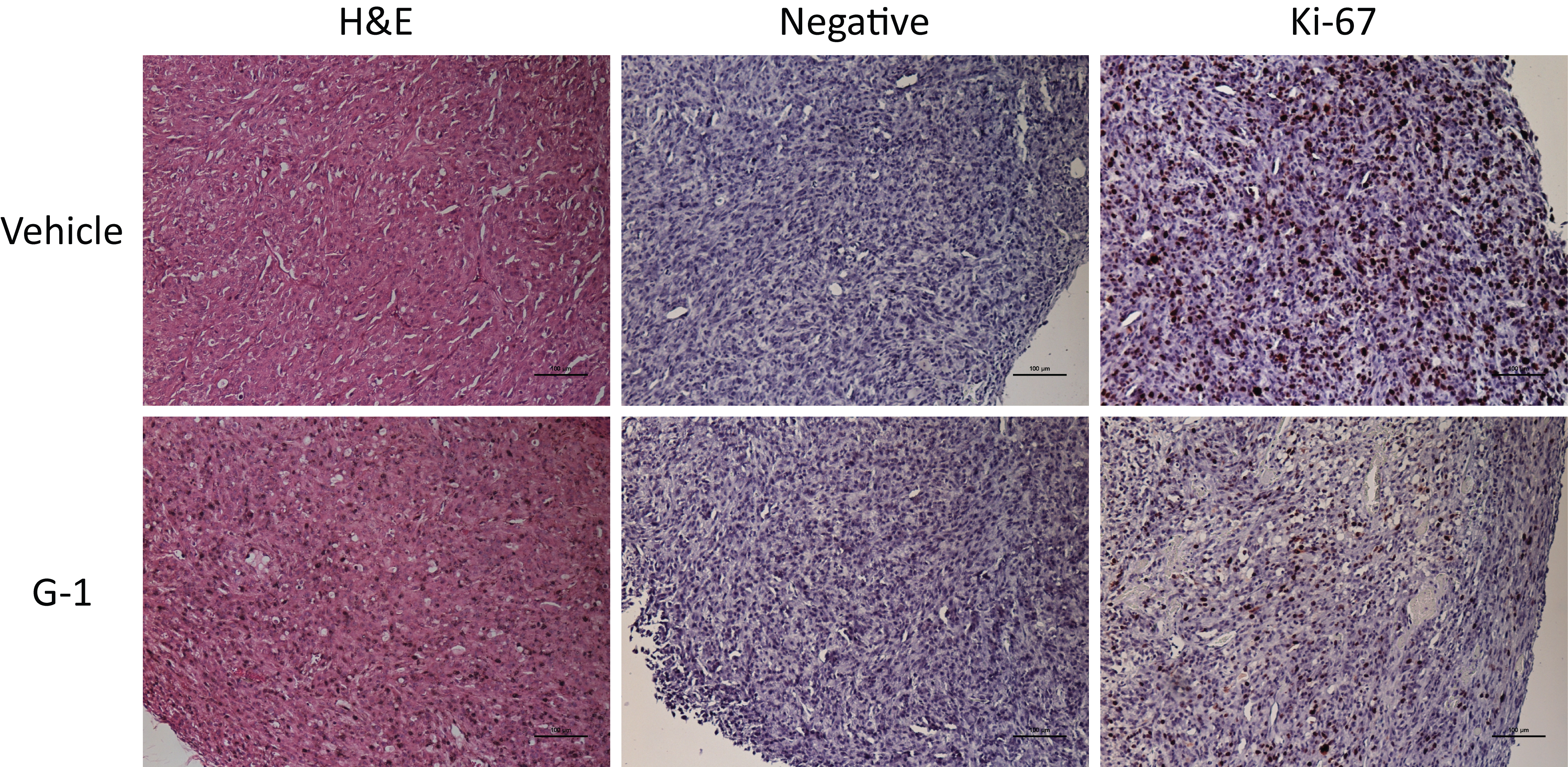

Supplement: Supplementary file 1 [file ijms-23-14309-s001.zip › Figure S3.png]

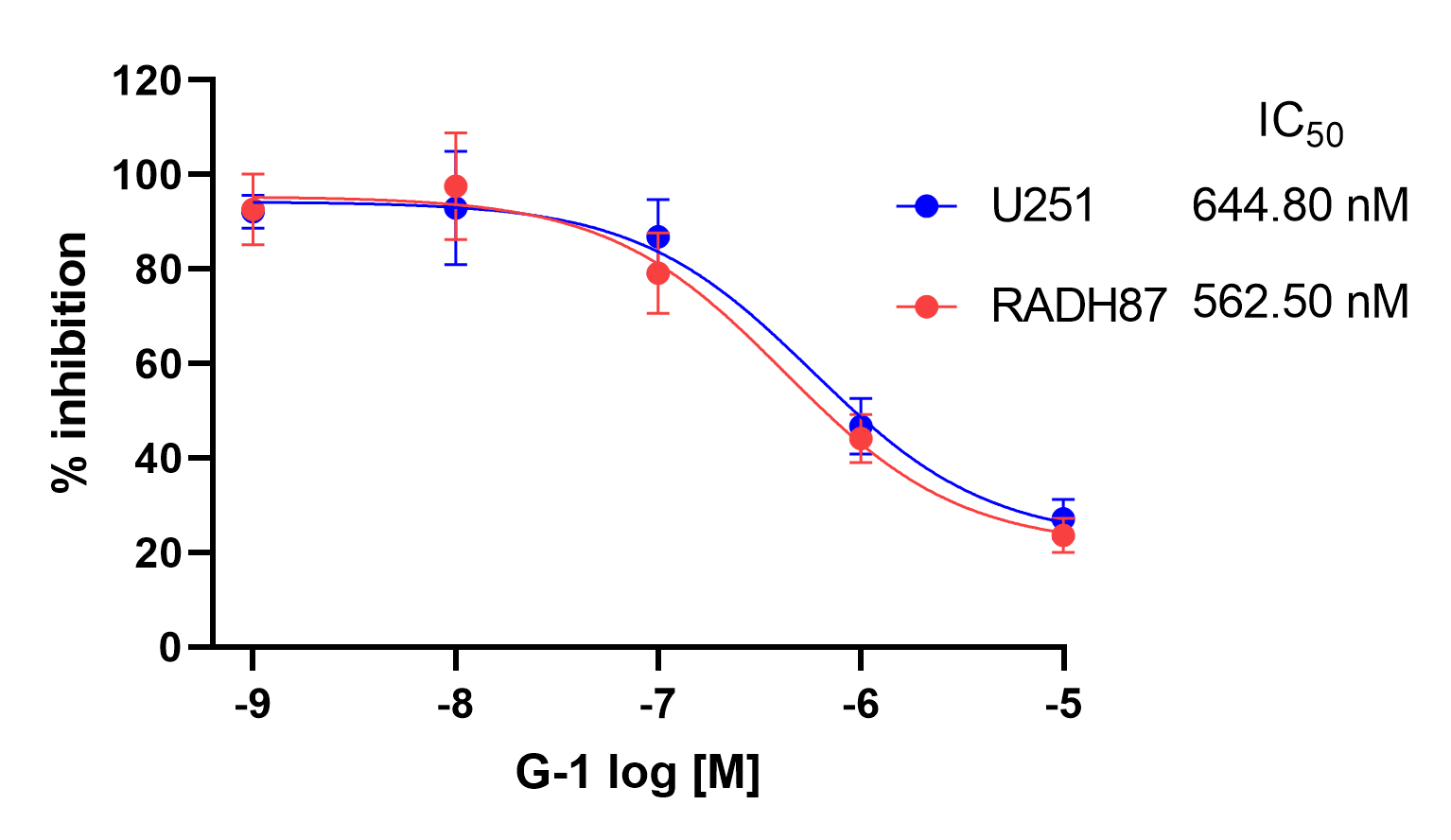

Supplement: Supplementary file 1 [file ijms-23-14309-s001.zip › Figure S4.png]
